# Simplifying Asthma: Understanding and managing asthma in young children in primary care

**DOI:** 10.12669/pjms.41.8.11769

**Published:** 2025-08

**Authors:** Syeda Ariba Hashmi, Munazza Asad

**Affiliations:** 1 Syeda Ariba Hashmi, FCPS Postgraduate Department of Family Medicine, Aga Khan University Hospital, Karachi, Pakistan; 2 Munazza Asad, Lecturer Department of Family Medicine, Aga Khan University Hospital, Karachi, Pakistan

**Keywords:** Asthma, Young children, CME, GINA, Primary care

## Abstract

Asthma is a common ailment presenting in primary care clinics, but it poses significant challenges in diagnosis and management for children under five years of age. Diagnostic tools like spirometry are not feasible for this age group and symptoms such as wheezing are common in many respiratory conditions. Treatment is equally challenging, as standard asthma medications cannot be administered in the same manner to young children as in adults and memorizing treatment guidelines can be difficult. To address this, we organized a Continuing Medical Education (CME) session featuring case-based discussions following a survey on asthma knowledge. This manuscript provides a concise summary of the activity, aiming to simplify asthma management for primary care physicians worldwide, particularly in children under five.

## INTRODUCTION

Asthma is a chronic inflammatory disorder of the airways characterized by episodes of reversible airway narrowing and obstruction, resulting in recurrent cough, wheeze, shortness of breath and chest tightness. It is a major respiratory problem in Pakistan, with an estimated 15 million children and 7.5 million adults affected.^1^ According to the global population data from the United Nations^2^ and Pakistan Bureau of Statistics,^3^ approximately 35-40% of Pakistan’s population is under the age of 18, with a significant proportion of children under the age of five years. The paediatric patient population presenting to primary care in Pakistan is substantial, with a high number of children requiring care for common illnesses and chronic conditions. Childhood asthma is amongst the most common chronic diseases in children and one of the main causes of missed school days. It tends to affect more male children than female.^4^ Its prevalence has increased substantially in recent years, placing a significant burden on affected children, their families and the healthcare system in Pakistan.^5^ Various factors contribute to its increasing incidence such as allergens, environmental pollution, urbanization, as well as genetic predisposition.

The severity ranges from intermittent symptoms to potentially life-threatening airway compromise, necessitating a comprehensive diagnostic approach and timely management. Most of these patients present to primary care clinics, where early recognition and intervention are crucial. Effective asthma management, however, remains a challenge for many healthcare providers.^6^ The complexities of paediatric asthma management stem not only from difficulties in its diagnosis but also from challenges in recalling and adhering to evidence-based management guidelines.

### Background:

Approach to a patient with asthma-like symptoms requires a thorough history taking and physical examination. Asthma-like symptoms including cough, wheeze and shortness of breath are common symptoms in several other conditions like bronchiolitis, pneumonia, allergic rhinitis, viral infections and even gastroesophageal reflux disease (GERD). The gold standard of diagnosis is spirometry which is recommended for patients aged five years or older, as younger individuals cannot perform this test.^7^ These overlapping symptoms and limited diagnostic modalities make asthma a diagnostic challenge. One of the key steps in tackling this dilemma is promoting continuing medical education (CME) programs focused on asthma management.

We arranged an educational session for physicians in the department of Family Medicine in a tertiary care hospital of Karachi, Pakistan. A total of 40 participants completed the survey—all physicians who attended the CME session were included. We presented a simple stepwise approach for primary care physicians for easy practical implications while dealing with such patients. The CME consisted of an online survey followed by a case-based presentation. In the online survey the participants were asked questions regarding childhood asthma to assess their knowledge and exposure to such patients. Almost half (50%) of the participants often see children with wheeze in their clinics while the rest get to see them occasionally.

Many participants (60%) chose ‘inhaled corticosteroids (ICS) plus as-needed short acting beta-agonist (SABA)’ as the best regime option for children with intermittent wheezing, while 30% opted for ‘as-needed SABA’ and 10% for ‘daily ICS’. We asked participants about the educational information patients need after using ICS with a facemask; 80% of the participants reported educating patients to rinse their mouth after taking ICS through a facemask while the correct response is to instruct patients to clean their face and nose. Mouth rinsing is recommended only when an inhaler is used through the mouth. When asked about the most effective regime for a child with wheeze and upper respiratory symptoms, 40% responded ‘as-needed SABA’, 35% responded ‘ICS as-needed’ and 25% responded with ‘daily ICS with as-needed SABA’.

### Stepwise Asthma Management:

The survey was followed by a case-based approach to educate physicians regarding the latest asthma management strategies in patients younger than five years of age. We presented a scenario showcasing history of asthma and the participants were asked to pick key features favoring asthma, which were cough, difficulty breathing, intermittent wheezing with physical activity, symptoms that got triggered with viral upper respiratory tract infection (URTI), worse symptoms at night and history of similar symptoms during seasonal variation. According to Global Initiative for Asthma (GINA)^8^ a child can be diagnosed with asthma if he had recurrent symptoms of wheezing or coughing that occurs with exercise (playing), laughing, crying, exposure to strong smell or in the absence of an apparent respiratory infection; nocturnal symptoms or awakening and presence of risk factors such as family history of asthma, atopy, allergic sensitization or a personal history of food allergy or atopic dermatitis.

The participants were then asked to pick factors responsible for poor prognosis of asthma, which included lack of immunization, exposure to dust or smoke, history of wheezing without colds, low birth weight,^9^ history of atopy, passive smoking, history of maternal smoking, parental asthma or family history of allergies. Other factors include obesity, chronic sinusitis, reflux, anxiety/depression, sleep apnea and rhinitis. Examination of the patients should always start with a general look, whether they are alert, oriented or drowsy and in distress. Other components of general physical examination include cyanosis, pallor and nasal flaring.

**Fig.1.1:**
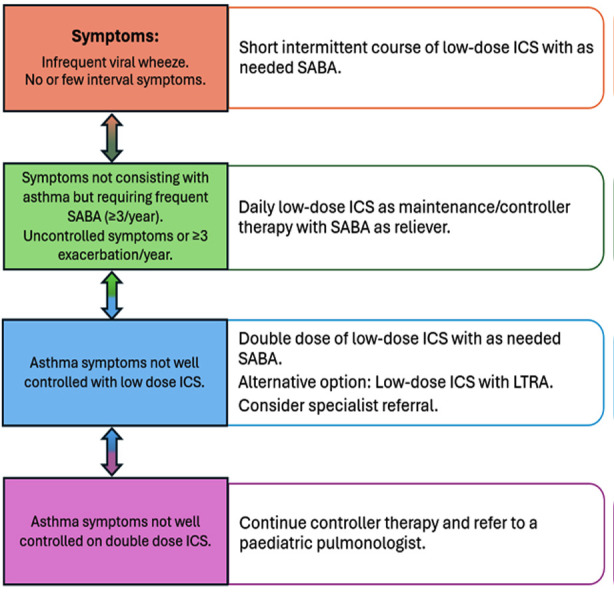
Asthma management steps based on symptom severity.

This is followed by measurements of vitals, especially respiratory rate (RR), oxygen saturation (SpO2), pulse and temperature. Chest examination includes inspection for intercostal or subcostal recessions and tracheal tug, followed by auscultation of bilateral lung fields. These steps are crucial in assessing the severity of asthma and steers the direction of our management accordingly. The presence of wheeze favours the diagnosis of asthma and signs of respiratory distress may suggest asthma exacerbation which will be discussed later. Table-I summarizes the approach to diagnosis of asthma. The goal of asthma management is to achieve and maintain good long-term control of symptoms, normal activity levels and minimize risk of flare-ups. The treatment follows a stepwise approach with medication adjusted up or down according to the symptoms (Fig.1.1). Children who experience infrequent viral-induced wheeze and no interval episodes should be provided with intermittent short course of ICS with as-needed SABA for relief of symptoms.

**Table-I:** Summary of important history and examination points of a young child with asthma.

Symptoms favoring asthma	• Recurrent history of cough, wheeze and shortness of breath during physical activity
	• Diurnal variation
	• Child’s history of atopy or eczema
	• Family history of asthma or atopy
	• Relief of symptoms after using inhaler (ICS or SABA)
	• Limitation of physical activity due to fatigue
Risk factors for persistence of childhood asthma	• Personal history of atopy
	• Parental asthma or atopy
	• Wheezing without URTI
	• Maternal smoking
	• Exposure to dust or smoke
Factors related to poor outcome of asthma	• Low birth weight (<24kg)
	• Passive smoking
	• Obesity
	• Rhinitis
	• Reflux
	• Vitamin D deficiency^10^
Examination	• Presence of signs of respiratory distress (tachypnea, nasal flaring, chest indrawing, subcostal or intercostal recessions, tracheal tug)
	• Wheeze on chest auscultation

The need for SABA reliever more than twice a week over a month period indicates escalating the medication to daily low-dose ICS with as-needed SABA as the preferred treatment. It should be given for at least three months to establish its effectiveness. Other less effective options include daily leukotriene receptor antagonist (LTRA) such as montelukast or short intermittent course of ICS whenever needed. Caregivers should be counselled regarding the potential adverse effect of montelukast on sleep and behaviour. If three months of daily low-dose ICS fails to control symptoms, then doubling the dose of ICS is the next best step. Adding LTRA to daily low-dose ICS can also be considered. If this fails to achieve good control, inhaler technique and medication adherence should be reassessed. In addition, specialist referral should be considered.

Symptom control and medication side-effects should be assessed in every visit. The child/ caregiver should be asked whether since the last visit they had:

1. Daytime asthma symptoms for more than a few minutes or more than once a week?
2. Any activity limitation due to asthma (runs less/ easily tires during playing)?
3. SABA reliever needed more than once a week.
4. Any night waking or nocturnal cough due to asthma?

Affirmative answer to all four or any three questions indicates uncontrolled symptoms and positive answer to one to two questions means partly controlled asthma, both requiring treatment escalation. Negative answer to all questions indicates well-controlled asthma hence the same treatment strategy should be continued for the next three months and de-escalation of medication can be considered on follow-up. Finally, non-pharmacological measurements should be addressed such as avoiding asthma triggers, optimizing weight and flu and pneumococcal vaccination.

### Asthma Exacerbation:

**Fig.1.2:**
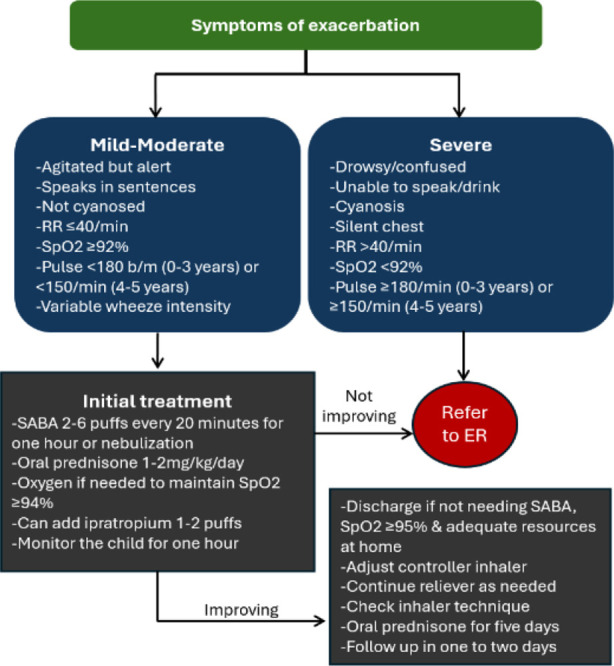
Asthma exacerbation management.

Early symptoms of exacerbation in young children include worsening cough especially at night, lethargy or impaired daily activity (including feeding) and no response to reliever medication. A quick examination must be done to assess the severity. Children with mild to moderate exacerbation are agitated but alert, breathless but able to speak in sentences with a RR of ≤40 breaths/minute (bpm), SpO2 ≥92%, pulse <180 beats/minute (b/m) (0-3 years) or <150 b/m (4-5 years) and variable wheeze intensity. This can be managed in a primary care setting with SABA 100mcg 2-6 puffs or 2.5mg by nebulizer (Fig.1.2). Repeat this after every 20 minutes for the first hour. Oxygen is given if needed to maintain SpO2 ≥94% and adding ipratropium 1-2 puffs can be considered. Reevaluate the child in one hour, and if their condition has improved, they may be discharged with controller inhaler, oral prednisone 1-2mg/kg/day for five days and early follow-up in 48 hours. If there is no improvement, then urgent transfer to a tertiary care setting is required. Children with severe exacerbation present with altered level of consciousness, unable to speak or drink, cyanosed with SpO2 <92%, RR >40 bpm, pulse ≥180 b/m (0-3 years) or ≥150 b/m (4-5 years) and silent chest on auscultation. Immediate transfer to a tertiary care setting is indicated, all the while providing inhaled or nebulized SABA and oxygen during the transfer.

## CONCLUSION

In primary care, the implementation of asthma guidelines faces significant challenges. Physicians often experience heavy workloads, resulting in limited opportunities to educate patients adequately. Furthermore, up-to-date clinical knowledge is not always accessible, and medication shortages—particularly in rural regions—compound these difficulties. To address these issues and improve adherence to guidelines, practical strategies might include incorporating visually engaging infographics, increasing the frequency and accessibility of CME sessions and making broader improvements to the primary healthcare infrastructure.

This method of teaching was welcomed by many participants as they were able to detect and rectify their areas of lacking. Through repeated exposure and reinforcement of knowledge with help of technology, healthcare providers can deepen and fortify their understanding of medical concepts more efficiently.

## References

[1] Khan MA (2022). Monthly and seasonal prevalence of asthma and chronic obstructive pulmonary disease in the District Dera Ismail Khan, Khyber Pakhtunkhwa, Pakistan. Egypt J Bronchol.

[2] United Nations Population Division (World Population Prospects):United Nations. Department of Economic and Social Affairs. Population Division (2022). World Population Prospects.

[3] Pakistan Bureau of Statistics:Pakistan Bureau of Statistics Population Census.

[4] Fuseini H, Newcomb DC (2017). Mechanisms driving gender differences in asthma. Current allergy and asthma reports.

[5] Rai VR (2022). Taking Control of the Breath:Prioritizing Childhood Asthma in Pakistan's Healthcare Agenda. Isra Med J.

[6] Mustafa G (2022). How pediatrician manage asthma in tertiary care hospitals of Multan, Pakistan- A cross sectional study. European Respir J.

[7] Lizzo JM, Goldin J, Cortes S (2025). Pediatric Asthma 2024 May 4. In:StatPearls.

[8] Global Initiative for Asthma (GINA) (2024). Global strategy for asthma management and prevention.

[9] Liu X, Olsen J, Agerbo E, Yuan W, Cnattingius S, Gissler M, Li J (2014). Birth weight, gestational age, fetal growth and childhood asthma hospitalization. Allergy Asthma Clin Immunol.

[10] Bantz SK, Zhu Z, Zheng T (2015). The role of vitamin D in pediatric asthma. Ann Pediatr Child Health.

